# An *Ixodes minor* and *Borrelia carolinensis* enzootic cycle involving a critically endangered Mojave Desert rodent

**DOI:** 10.1002/ece3.957

**Published:** 2014-01-30

**Authors:** Janet Foley, Caitlin Ott-Conn, Joy Worth, Amanda Poulsen, Deana Clifford

**Affiliations:** 1 Department of Medicine and Epidemiology, School of Veterinary Medicine, University of California1320 Tupper Hall, Davis, California, 95616; 2 Wildlife Investigations Lab, California Department of Fish and Wildlife1701 Nimbus Road, Rancho Cordova, California, 95670; 3 Wildlife Health Center, University of CaliforniaDavis, California, 95616

**Keywords:** 16S rRNA, Amargosa vole, *Borrelia burgdorferi* sensu lato, calreticulin, *Microtus californicus scirpensis*, Migration

## Abstract

*Microtus californicus scirpensis* is an endangered, isolated subspecies of California vole. It requires water pools and riparian bulrush (*Schoenoplectus americanus*) and occupies some of the rarest habitat of any North American mammal. The minimally vegetated, extremely arid desert surrounding the pools is essentially uninhabitable for *Ixodes* species ticks. We describe an enzootic cycle of *Borrelia carolinensis* in *Ixodes minor* ticks at a site 3500 km distant from the region in which *I. minor* is known to occur in Tecopa Host Springs, Inyo County, eastern Mojave Desert, California. Voles were live-trapped, and ticks and blood samples queried by PCR and DNA sequencing for identification and determination of the presence of *Borrelia* spp. Between 2011–2013, we found 21 *Ixodes minor* ticks (prevalence 4–8%) on Amargosa voles and *Reithrodontomys megalotis*. DNA sequencing of 16S rRNA from ticks yielded 99% identity to *I. minor*. There was 92% identity with *I. minor* in the *calreticulin* gene fragment. Three ticks (23.1%), 15 (24%) voles, three (27%) house mice, and one (7%) harvest mice were PCR positive for *Borrelia* spp. Sequencing of the 5S-23S intergenic spacer region and *flagellin* gene assigned Amargosa vole *Borrelia* strains to *B. carolinensis. Ixodes minor*, first described in 1902 from a single Guatemalan record, reportedly occurs only in the southeast American on small mammals and birds. The source of this tick in the Mojave Desert and time scale for introduction is not known but likely via migratory birds. *Borrelia* strains in the Amargosa ecosystem most closely resemble *B. carolinensis. B. carolinensis* occurs in a rodent-*I. minor* enzootic cycle in the southeast U.S. although its epidemiological significance for people or rodents is unknown. The presence of a tick and *Borrelia* spp. only known from southeast U.S. in this extremely isolated habitat on the other side of the continent is of serious concern because it suggests that the animals in the ecosystem could be vulnerable to further incursions of pathogens and parasites.

## Introduction

The Amargosa vole (*Microtus californicus scirpensis,* Kellogg, 1918) is a critically endangered and very isolated subspecies of California vole found only in the eastern Mojave Desert in Inyo County, California (U.S. Fish and Wildlife Service 1997; Cudworth and Koprowski 2010). This approximately 75 g microtine rodent requires riparian habitat dominated by bulrush (*Schoenoplectus americanus,* (Persoon) Volkart, 1905) and thus has one of the narrowest niche breadths and occupies some of the rarest habitat of any North American mammal. Suitable, occupied habitat occurs at present only within the Amargosa River basin near the town of Tecopa and is surrounded by harsh, alkali desert.

Federal protection under the U.S. Endangered Species Act for the Amargosa vole was motivated by habitat loss and degradation, with very low population size, habitat fragmentation, genetic impoverishment, and disease all cited as contributory factors (U.S. Fish and Wildlife Service 1997). Since 2011, a collaborative group of researchers has begun to catalog pathogens and ectoparasites of the Amargosa vole and sympatric small mammals, initially identifying trombiculid mite larvae as a cause of severe ear tissue destruction in many vole individuals (Foley et al. 2013). In the course of this survey, we also obtained ixodid ticks from some of the voles and detected *Borrelia burgdorferi* (Johnson et al., 1984) sensu lato DNA from ear tissue of individuals. In California, *Borrelia burgdorferi* sensu stricto appears to be the most significant agent of Lyme disease and its reservoirs are small mammals such as the western gray squirrel (*Sciurus griseus,* Ord, 1818) (Lane et al. 2005). A cluster of genospecies designated *B. burgdorferi* sensu lato occurs in various small mammals but most, with the exception of *B. burgdorferi* sensu stricto*,* do not cause human Lyme disease (Girard et al. 2011). The ticks on the Amargosa voles, including adult stage ticks, appeared morphologically consistent with *Ixodes pacificus* (Cooley and Kohls, 1943)*,* a species that is the main vector for *B. burgdorferi* in the western U.S. (Burgdorfer et al. 1985; Clover and Lane 1995) but is extraordinarily rare as an adult on small mammals and never occurs at the low elevation, extremely arid, and unforested site occupied by the Amargosa vole (Furman and Loomis 1984). Because of this, we pursued further evaluation of the ticks and spirochetes including molecular characterization. In the present study, we describe an enzootic cycle of *B. carolinensis* (Rudenko et al. 2011) in *I. minor* (Neumann 1902) ticks at a site 3500 km distant from the region in which *I. minor* is known to occur.

## Materials and Methods

Small mammals were trapped in Sherman (HB Sherman, Tallahassee, FL) live traps along the Amargosa River in the vicinity of Tecopa Hot Springs in southeastern Inyo County, California (UTM Zone 11 N 3969717-3971539 and W 568016-569168) between November 2011 and February 2013. In these sites, riparian vegetation extends for only a few meters beyond river or pool margins and predominantly consists of bulrush (*Schoenoplectus americanus*) interspersed with cattails (*Typha domingensis,* Persoon, 1807), desert salt grass (*Distichlis spicata,* (L.) Greene, 1887), and rushes (*Juncus* spp.). Appropriate biosecurity was implemented including sterilization of traps, footwear, and equipment among sites. Traps were set at dusk and baited with peanut butter and oats and then animals recovered at dawn. Captured individuals were identified to species, sex, and age class, then weighed, evaluated for reproductive condition, and sampled for parasites and pathogens. Based on physical examination, animals were assigned a body condition score from 1 to 5 (Ullman-Cullere and Foltz 1999), all ectoparasites were collected into 70% ethanol, and a piece of marginal pinna was collected with a sterile scissors. Animals were then given a uniquely numbered metal ear tag (1005-1 Monel, National Band and Tag Co., Newport, KY) and released at the capture site. Each successful capture event was localized with a handheld global positioning system device (Garmin 62S, Garmin International, Olathe, KS). All work with animals was performed in compliance with the UC Davis IACUC and overseen by the campus attending veterinarian, under valid scientific collecting permits from California Department of Fish and Wildlife, US Fish and Wildlife Service, and Bureau of Land Management.

Initially, we attempted to identify *Ixodes* spp. ticks to species using keys (Furman and Loomis 1984; Webb et al. 1990) with larvae viewed under a compound microscope in a depression slide as well as a dissecting microscope before identification was confirmed. For further evaluation of tick morphology, we utilized an expanded set of resources from east of the Rocky Mountains (Cooley and Kohls 1945; Clifford et al. 1961; Keirans and Clifford 1978). DNA was extracted from ticks using ammonium hydroxide, modified from Humair et al. (2007). Ticks were individually placed in microcentrifuge tubes, cooled in liquid nitrogen for 3 min, and crushed using a microcentrifuge pestle. 100 mL of 0.7 mol/L NH_4_OH was added, and samples were placed on a 100°C heat block for 15 min. Tubes were then cooled on ice for 30 sec followed by an additional 15 min of heating at 100°C with open lids in order to evaporate the ammonia. In order to confirm tick identification, the 16S rRNA gene of the tick mitochondrion was amplified as previously described with modifications (Black and Piesman 1994) from four randomly chosen ticks using primers 16S+1 and 16S−2 to produce a 287-base-pair product. The 25 *μ*L PCR mix contained GoTaq Green Master Mix (Promega, Madison, WI), 0.5 *μ*mol/L of the forward primer, 0.5 *μ*mol/L of the reverse primer, and 1 *μ*L of DNA, and run conditions were as described in the original publication. A new PCR for a 242-base-pair fragment of the *calreticulin* gene was designed by hand after obtaining all sequences of *I. pacificus* and *I. minor calreticulin* fragments available on GenBank. The assay used forward primer 5′-GACGGAGGTAAGCCCCATTTTC-3′ and reverse primer 5′-GAACTTGCCGGCGGACAGCTTG-3′, mastermix as described for the 16S PCR, and the following run conditions: after denaturation at 94°C for 5 min, 30 cycles of 94°C for 30 sec, 58°C for 15 sec, and 72°C for 30 sec, with a final extension of 72°C for 1 min. Following PCR and agarose gel electrophoresis, DNA was purified from gels using a kit (QiaQuick, Qiagen, Valencia, CA) and submitted for sequencing on an ABI 3730 sequencer (Davis Sequencing, Davis, CA) using the forward PCR primer. Sequenced amplicons were evaluated by BLAST search of GenBank (NCBI; http://blast.ncbi.nlm.nih.gov/Blast.cgi). For final confirmation and for submission to the GenBank database, PCR amplicons were gel-extracted and then cloned using the pGEM-T easy vector system (Promega) followed by DNA sequencing using plasmid primer T7.

Ear tissue was kept frozen at −20°C, and then, DNA extraction carried out using the Qiagen Purification kit, following manufacturer's guidelines. TaqMan real-time PCR for *Borrelia* spp. was performed on ear and tick DNA as described previously (Barbour et al. 2009), modified to use only the forward and reverse primers, and the probe identified as specific for *B. burgdorferi* (although *in silico* analysis, our internal unpublished data show that both primers are generic for borreliae, while the probe has 100% homology to most if not all *B. burgdorferi* sensu lato genospecies including *B. bissettii, B. garinii,* and others). Reactions were run in a combined thermocycler/fluorometer (ABI Prism 7700, Applied Biosystems, Foster City, CA). Water negative controls were included in each run, while a panel of positive controls consisted of DNA from cultured strains of *B. burgdorferi* sensu stricto and *B. bissettii*. Strongly, PCR-positive samples with a cycle threshold <35 were evaluated using conventional PCR for three additional targets. 5S-23S intergenic spacer sequence (IGS) and *flagellin* protocols were performed as described with modifications to use GreenGoTaq Master Mix (Rudenko et al. 2011). PCR for the *Borrelia* 16S gene was carried out as described (Rudenko et al. 2011) with the following modifications: Amplitaq Gold Mastermix 360 (Life Technologies, Carlsbad, CA) was used in a 50 *μ*L reaction volume containing 1.0 *μ*mol/L primers and 10 *μ*L DNA. Initial denaturation was changed to 10 min at 96°C to initiate hotstart, and the rest of the cycle conditions remained the same as initially published. Amplicons were excised, cloned, and sequenced as for tick products. Tick 16S and *calreticulin* and *Borrelia* 16S, *fla*, and 5S-23 IGS sequences were deposited in GenBank, accession numbers (to be inserted).

## Results

Between autumn 2011 and spring 2013, we assessed 62 individual Amargosa voles, 15 harvest mice (*Reithrodontomys megalotis* (Baird, 1857)), and 11 house mice (*Mus musculus,* L., 1758) in marshes near Tecopa, CA. All ticks (*N* = 21) were morphologically confirmed as *I. minor*, differentiated from *I. pacificus* primarily on the basis of overall size and shape of the auriculae. All of the ticks except two on harvest mice were from voles. There were 13 adults (nine female and four male), three larvae, and five nymphs. The harvest mouse ticks were collected in October 2012, while the other ticks were collected in February (larvae, nymphs, and adults), March (nymphs and adults), and April (larvae). DNA sequencing from 16S rRNA from an *I. minor* larva, nymph, and a male and female adult yielded complete coverage of the 287 nucleotides, each with 99% identity to *I. minor* accession AF549841.1 in the GenBank database and only 91% identity with the next closest match (*I. muris,* Bishopp and Smith, 1937). All *I. minor* were identical to each other in the 16S gene. There was 92% identity with *I. minor* accession A4395264.1 in the *calreticulin* fragment, compared with 89% for *I. muris* and *I. jellisoni* (Cooley and Kohls, 1938) and 88% for *I. pacificus*.

**Figure 1:**
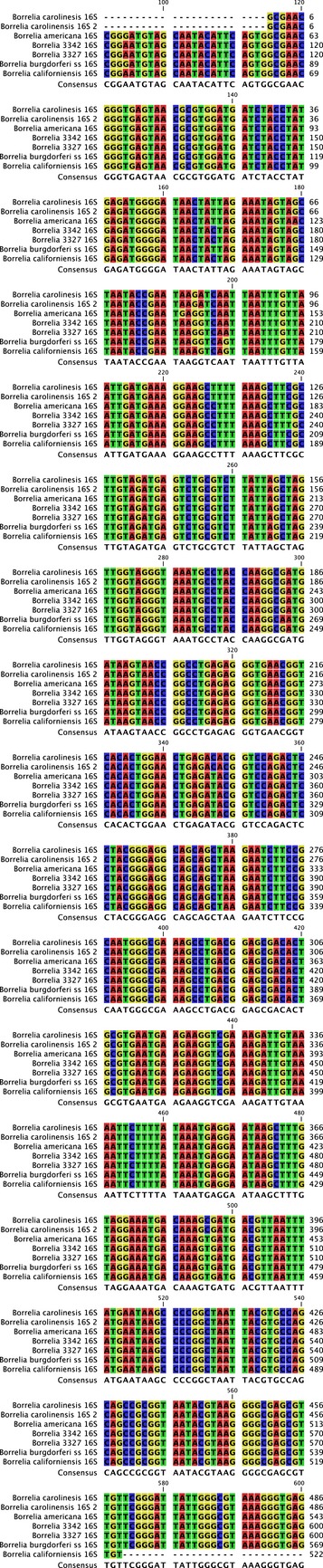
Multiple-sequence alignment for *Borrelia* genospecies from Amargosa vole tissue with other well-characterized *Borrelia* accessions from GenBank in the 16S rDNA. Amargosa vole sequences are designated 3327 and 3342, GenBank accession numbers in process. Other GenBank accessions are as follows: *B. carolinensis* strain SCCH-11, EU085416.1; *B. carolinensis* strain SCW-19, EU085409.1; *B. americana* strain SCW-41, EU081285.1; *B. burgdorferi* sensu stricto strain B31, AE000783.1; *B. californiensis* strain CA 443, DQ393303.1.

Of the ticks and rodents tested by PCR for *Borrelia* spp., three ticks (of 13, 23.1%) were positive, while 15 of 62 voles (24.2%), three of 11 house mice (27.3%) and one of 15 harvest mice (6.7%) were positive. Results of DNA sequencing showed that Amargosa vole *Borrelia* strains were closely related to several species of *B. burgdorferi* sensu lato. Sequencing of the 5S-23S IGS assigned Amargosa vole *Borrelia* strains to *B. carolinensis*. Three samples from Amargosa voles had identical sequences over the 253 base pairs and were 100% homologous with 15 different *B. carolinensis* strains in the database (e.g., strain SCSC-1, EU072440.1) and only 95% homology with the next closest match, *B. garinii*. In the *fla* gene, four amplicons had identical sequences and, over 494 base pairs, had 99% homology with multiple *B. carolinensis* accessions including strain SCCH-6, SCGT-18, and SCGT-21 (EU076485.1, EU076498.1, and EU076499.1), while the relatedness to *B. americanum* was only 97% and other genospecies were lower. For the 16S rRNA gene, PCR from two individuals yielded invariant sequences, but there were scattered differences across the fragment with sequences in GenBank and no consistently best match (Fig. 1). Rather, across the 18 single nucleotide polymorphisms (SNP) observed when vole sequences were aligned with well-defined sequences from multiple *B. americana, B. californiensis, B. carolinensis, B. bissettii,* and *B. burgdorferi* sensu stricto*,* two SNPs were unique to vole strains, two specifically matched only *B. californiensis,* while four ruled out *B. californiensis* (but did not distinguish among the other genospecies), four ruled out *B. americana,* four ruled out *B. carolinensis,* and two ruled out *B. burgdorferi* sensu stricto. Thus, overall Amargosa vole *Borrelia* strains appear to be more closely related to *B. carolinensis*.

## Discussion

South of Shoshone, California, the Amargosa River comprises stretches of southward-flowing water, subterranean flows, and occasional pools and springs until it finally makes a sharp turn northwest and terminates in Death Valley. While much of the riparian habitat along these waterways may have been occupied historically by Amargosa voles, all but the Tecopa population have been extirpated. Beyond the narrow strip of bulrush that fringes a few of the Amargosa pools near Tecopa, the eastern Mojave ecosystem is minimally vegetated, has a mean annual rainfall of only about 12 cm, and has a substrate of salt-encrusted alkaline clay flats (Williams et al. 1984). This landscape, by virtue of extreme aridity and lack of vegetated microhabitats, is essentially uninhabitable for *Ixodes* species ticks. Thus, an established colony of *I. minor* in a relict rodent population was a very unexpected finding.

*Ixodes minor*, first described in 1902 from a Guatemalan collection of a “*Hesperomys”* (possibly *Calomys* sp.)(Neumann 1902), was considered an invalid species in 1945 because of the lack of corroborating data (Cooley and Kohls 1945), rediscovered in Georgia as *I. bishoppi* (Smith and Gouck 1947), and then re-established with *I. bishoppi* as a junior synonym in 1961 (Clifford et al. 1961). The species is well documented from the 20th century in the American southeast from Florida to South Carolina, where it reportedly feeds in all stages primarily on small mammals, ground-feeding birds, and from a report on the eastern spotted skunk (*Spilogale putorius,* L, 1758) (Keirans and Clifford 1978; Tedders et al. 1992). This species rarely bites humans (Oliver et al. 2003). While *I. minor* and *I. pacificus* are both in the same subgenus (*Ixodes*), analysis of the 16S shows them to be in separate clades (Xu et al. 2003). Morphological characteristics such as size may allow for confirmation of species, but few resources directly compare *I. pacificus* with *I. minor,* and thus, we performed molecular analysis to confirm the species, to initialize a database from this western population, and to determine similarity with eastern specimens. We used the commonly reported 16S fragment but also chose the *calreticulin* gene, which at the time of the study was the only other gene reported in GenBank from both *I. pacificus* and *I. minor*. This analysis confirms the Amargosa vole tick as *I. minor* or a very close, not previously described relative.

We know little about the ecology of *I. minor* in the Amargosa ecosystem. In the American southeast, reports document adult activity in summer and October, nymphs during November and early summer, and larvae in November (Banks et al. 1998). In Tecopa however, trapping was restricted to months when ambient temperature does not endanger the voles in traps. House mice (*Mus musculus*) are infested in the eastern U.S. and have been introduced into some of the Tecopa marshes. It would be valuable to assess small mammals from all potential vole habitat near Tecopa for this tick and such studies are underway. The source of this tick into the Mojave Desert and time scale for its introduction are not known. As small mammals generally are not particularly vagile, a more plausible source is on migratory birds; indeed, movement on migratory birds of ticks including *I. minor* onto barrier islands in the southeast has been hypothesized (Wilson and Durden 2003). However, migration routes tend to be along north-south flyways, and birds would be unlikely to travel from Georgia or Florida directly to southern California. Rather, we posit that some individuals may have migrated from southeastern North America to Mexico or Central America, with occasional movement of birds not back to the eastern U.S. but rather up the west coast flyway. If our hypothesis is accurate, it suggests two important directions for further investigation. Of the two large tick studies from Mexico and Central America, neither the tick fauna from Panama nor Mexico list this species (Hoffmann 1962); however, Mexican and Central American regions where birds overwinter should be further evaluated for this tick, as it was first found in Guatemala and its migration may be an important piece of the puzzle of its ecology. Additionally, Tecopa marshes are a very small (albeit important) target for northward migrating birds and their ticks: if *I. minor* were introduced via this migration route, it would seem even more probable that other larger suitable habitat patches in the western U.S. might be targets as well, and perhaps, *I. minor* has been overlooked or mis-identified as *I. pacificus* elsewhere.

The *B. burgdorferi* sensu lato group of related strains and genospecies has experienced considerable revision, with discovery of new strains as well as phylogenetic and ecological studies that reveal significant distinctions among the strains. However, without full genome sequencing of multiple replicates, coupled with extensive ecological studies to understand host and vector extent and competence for each of the various genospecies, species boundaries seem slightly insecure at present, especially given that the GenBank database is full of older deposited material with nomenclature reflecting the designation at the time of submission rather than updated species names. Nevertheless, comprehensive comparison of *fla,* 16S, and 5S-23S IGS sequences from *Borrelia* strains in the Amargosa ecosystem to accessions that were recent and well supported in the literature revealed that Amargosa strains most closely resemble *B. carolinensis. B. carolinensis* was formally described in 2011 and has been cultured from the cotton mouse (*Peromyscus gossypinus,* (Le Conte, 1850) and eastern woodrat [*Neotoma floridana,* (Ord, 1818)] and from an *I. minor* on a woodrat in South Carolina (Rudenko et al. 2011). It has not previously been reported from outside the American southeast and has unknown epidemiological significance for humans or rodents. It is closely related to *B. bissettii,* which is present in California and has been associated with human Lyme disease (Rudenko et al. 2009). Oliver et al. (2003) reported enzootic maintenance of *B. bissettii*-like bacteria in *I. minor* and small mammals in the southeastern U.S. Very recent research has expanded our understanding of the pathogenic potential for *B. burgdorferi* and related genospecies such as *B. miyamotoi* and *B. americanum* (Clark et al. 2013; Krause et al. 2013), suggesting further consideration of disease association for *B. carolinensis* is warranted.

The Amargosa vole, with its dependence on very limited, patchily-distributed, and highly specialized habitat, is at considerable danger of extinction. Endemic diseases may fail to persist in such small patches, and the remoteness of the ecosystem would tend to limit the introduction of pathogens. However, we show that a tick and bacterium from the farthest possible side of the continent are relatively common in Amargosa voles. It is not known when these organisms were introduced, although further molecular study could reveal their phylogeographic history. Their presence is of serious concern because it suggests that the animals in the ecosystem could be vulnerable to further incursions of pathogens and parasites. In addition to the multiple infectious challenges to which these voles already are exposed, introductions of non-native disease must also be identified and managed in order to contribute to a realistic recovery plan for the species.
